# Duodenal endoscopic submucosal dissection using dual-channel rapid hemostasis for a large tumor involving the papilla

**DOI:** 10.1055/a-2709-7362

**Published:** 2025-10-13

**Authors:** Tatsuma Nomura, Makoto Kobayashi, Junya Yamada, Hiroaki Kumazawa, Yoshiaki Isono, Katsumi Mukai

**Affiliations:** 1 Department of Gastroenterology, Suzuka General Hospital, Suzuka, Mie, Japan; 2 Department of Endoscopy Center, Suzuka General Hospital, Suzuka, Mie, Japan; 3 37036 Department of Gastroenterology, Yokkaichi Municipal Hospital, Yokkaichi, Mie, Japan

Duodenal endoscopic submucosal dissection (ESD) is a challenging procedure for large tumors involving the papilla 1 . We previously reported a dual-channel rapid hemostasis (RH) technique using a gas-free immersion (GFI) system that effectively stopped bleeding during gastric ESD with saline immersion 2 3 . This method involves performing ESD using a double-channel endoscope (GIF-2TQ260M; Olympus), with hemostatic forceps pre-inserted into one of the accessory channels. Here, we present a case of a large duodenal tumor involving the papilla, in which ESD was performed using this method.

The patient had a 50-mm duodenal tumor in the papilla. En bloc resection was performed using saline-immersion ESD with RH and the GFI system ( Fig. 1 , Video 1 ). First, a calibrated, small-caliber tip, transparent hood (CAST hood; TOP, Tokyo, Japan) with a 4-mm tapered tip was attached to the scope 4 . Hemostatic forceps were preinserted into one of the accessory channels. After insertion of the scope, a hyaluronic acid solution was locally injected into the submucosal layer, and a mucosal incision was made at a distance from the tumor. The papilla was intentionally preserved, and an incision was made directly above the muscle layer to separate the bile and pancreatic ducts. A large blood vessel was observed on the anal side of the papilla and was pre-sealed with hemostatic forceps. When active bleeding occurred during submucosal dissection, hemostasis was easily achieved by bringing the hemostatic forceps close to the bleeding point and closing them. Flow-assisted coagulation using GFI enabled non-contact hemostasis under saline immersion by positioning the forceps near the bleeding point and coagulating the tissue. Using these techniques, the tumor was resected in 43 minutes. The remaining mucosal defect measured 60 mm; therefore, endoscopic biliary and pancreatic drainage tubes were inserted, and the defect was completely closed using the reopenable clip over the line method 5 .

**Fig. 1:**
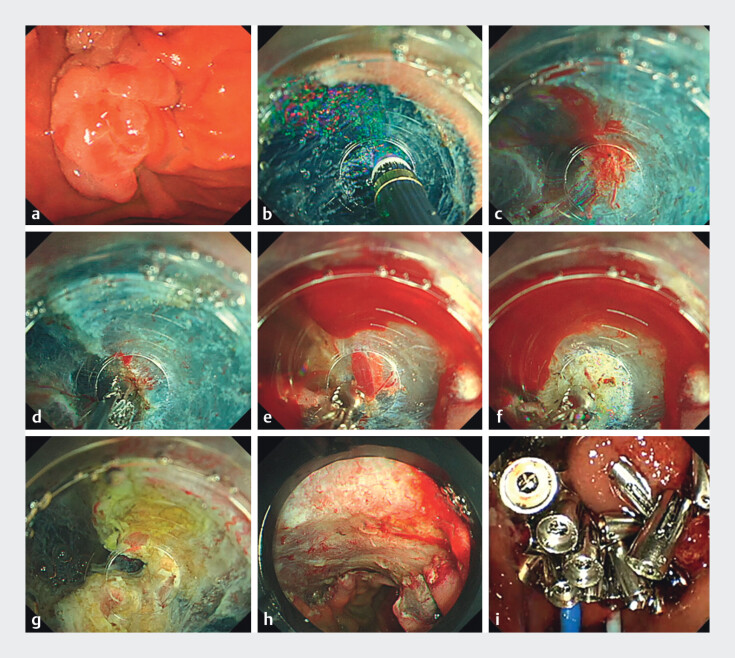
Endoscopic submucosal dissection (ESD) of a large duodenal tumor involving the papilla using dual-channel rapid hemostasis and the gas-free immersion (GFI) system. **a** Duodenal tumor including the papilla, measuring 50 mm. **b** Submucosal dissection using the GFI system with a calibrated, small-caliber tip, transparent hood (CAST hood) featuring a 4 mm a tapered tip, enabling dissection without visual interruption from bubbles. **c, d** In case of bleeding, rapid hemostasis can be achieved by compressing the bleeding site with the broad surface of the CAST hood. **e, f** Bleeding from thick blood vessels is stopped using flow-assisted coagulation with the GFI system, achieving hemostasis across an area matching the inner diameter of the CAST hood. **g** The bile and pancreatic ducts were separated just above the muscle layer, allowing direct visualization of both openings. **h** A 60 mm mucosal defect following complete resection, including the papilla. **i** Complete closure of the mucosal defects using the reopenable clip over the line method, with endoscopic biliary and pancreatic drainage tubes inserted without embedding.

Duodenal ESD of a large tumor involving the papilla using dual-channel rapid hemostasis and the gas-free immersion system.Video 1

Endoscopy_UCTN_Code_TTT_1AO_2AG_3AD

## Notes

Endoscopy E-Videos https://eref.thieme.de/e-videos
*E-Videos* is an open access online section of the journal *Endoscopy* , reporting on interesting cases and new techniques in gastroenterological endoscopy. All papers include a high-quality video and are published with a Creative Commons CC-BY license. Endoscopy E-Videos qualify for HINARI discounts and waivers and eligibility is automatically checked during the submission process. We grant 100% waivers to articles whose corresponding authors are based in Group A countries and 50% waivers to those who are based in Group B countries as classified by Research4Life (see: https://www.research4life.org/access/eligibility/ ). This section has its own submission website at https://mc.manuscriptcentral.com/e-videos .
